# Effect of 12-month intervention with lipid-based nutrient supplement on the physical activity of Malawian toddlers: a randomised, controlled trial

**DOI:** 10.1017/S0007114517000290

**Published:** 2017-02-28

**Authors:** A. Pulakka, Y. B. Cheung, K. Maleta, K. G. Dewey, C. Kumwenda, J. Bendabenda, U. Ashorn, P. Ashorn

**Affiliations:** 1 Department of Public Health, University of Turku and Turku University Hospital, 20014 Turku, Finland; 2 Centre for Quantitative Medicine, Duke-National University of Singapore Medical School, Singapore 169857, Singapore; 3 Department of Biostatistics, Singapore Clinical Research Institute, Singapore 138669, Singapore; 4 School of Public Health and Family Medicine, University of Malawi College of Medicine, Mahatma Gandhi Road, Blantyre, Malawi; 5 Department of Nutrition, University of California, Davis, Davis, CA 95616-5270, USA; 6 Department of Human Nutrition and Health, Lilongwe University of Agriculture and Natural Resources, PO Box 219, Lilongwe, Malawi; 7 Tampere Center for Child Health Research, University of Tampere and Tampere University Hospital, 30014 Tampere, Finland; 8 Department of Paediatrics, Tampere University Hospital, 33521 Tampere, Finland

**Keywords:** Lipid-based nutrient supplements, Undernutrition, Physical activity, Accelerometer, Sub-Saharan Africa

## Abstract

Physical activity is beneficial for children’s well-being. The effect of dietary supplementation on children’s physical activity in food-insecure areas remains little studied. We examined the effects of a lipid-based nutrient supplement (LNS) on children’s objectively measured physical activity in a randomised, controlled, outcome-assessor-blinded trial. Mothers of the children received one capsule daily of Fe-folic acid (IFA), one capsule containing eighteen micronutrients (MMN) or one 20 g sachet of LNS (containing twenty-two MMN, protein, carbohydrates, essential fatty acids and 494 kJ (118 kcal)) during pregnancy and for 6 months thereafter. Children in the IFA and MMN groups received no supplementation, and these groups were collapsed into a single control group; children in the LNS group received 20 g LNS from 6 to 18 months. We measured physical activity with accelerometers over 1 week at 18 months. The main outcome was mean vector magnitude counts/15 s. Of the 728 children at the beginning of child intervention at 6 months, 570 (78 %) provided sufficient data for analysis. The mean accelerometer counts for the 190 children in the LNS group and for the 380 children in the control group were 303 (sd 59) and 301 (sd 56), respectively (*P*_for difference_=0·65). LNS, given to mothers during pregnancy and 6 months postpartum and to their infants from 6 to 18 months of age, did not increase physical activity among 18-month-old children.

Studies in Ghana^(^ ^1^ ^,^ ^2^ ^)^, Malawi^(^ ^3^ ^,^ ^4^ ^)^, Haiti^(^ ^5^ ^)^ and Burkina Faso have tested the growth-promoting efficacy of small-quantity, lipid-based nutrient supplements (LNS) as a low-cost solution for tackling growth-faltering in young children. The studies have demonstrated modest^(^ ^1^ ^,^ ^2^ ^,^ ^5^ ^)^ or no effects^(^ ^3^ ^,^ ^4^ ^)^ of LNS supplementation on child growth. At least three possible explanations for limited growth response to supplementation exist. First, supplementation starting at 6 months of age might come too late as children in resource-poor settings are commonly affected by intra-uterine growth restriction and thus are born stunted^(^ ^6^ ^)^. Second, subclinical infection or inflammation may block the growth response to nutrient supplements, suggesting that interventions combining nutrition interventions with prevention and management of infections might increase the effect^(^ ^6^ ^–^ ^8^ ^)^. Third, energy from the supplementary food may be diverted to other needs of the children, such as physical activity^(^ ^9^ ^,^ ^10^ ^)^.

Physical activity, which in young children is exhibited commonly in the form of play^(^ ^11^ ^)^, is an important determinant of young children’s cognitive and motor development^(^ ^12^ ^,^ ^13^ ^)^ and a contributor to their health in later life^(^ ^14^ ^,^ ^15^ ^)^. Some studies have found that undernourished children are less physically active than well-nourished children, but increase their activity level as their nutritional status improves^(^ ^16^ ^–^ ^18^ ^)^. This is plausible, given that the human body undergoes a series of physiological and behavioural changes, including reduction of physical activity, as a response to lowered energy intake^(^ ^19^ ^,^ ^20^ ^)^. In addition to energy deficiency, micronutrient deficiencies, especially Fe deficiency, have been shown to result in reduced activity levels^(^ ^21^ ^–^ ^24^ ^)^. However, there is limited information on physical activity, measured by modern, objective methods^(^ ^18^ ^,^ ^24^ ^–^ ^26^ ^)^, of young children in resource-poor settings, although some studies from high-income countries exist^(^ ^27^ ^–^ ^29^ ^)^.

Reduced physical activity of undernourished children is of concern, because it can be one of the mediators leading to poor child development. This theory is known as the functional isolation hypothesis: behaviour of undernourished children – that is, reduced activity and exploration and increased apathy – results in children having less interaction with their environment, and this in turn may restrict optimal development^(^ ^23^ ^,^ ^30^ ^)^. Moreover, in response to the undernourished children’s behaviour, caregivers may offer them less stimulation, which again hinders their development^(^ ^16^ ^,^ ^30^ ^)^.

Studies on the effect of nutrient supplementation on physical activity, using accelerometer-measured counts as an outcome, in areas with high prevalence of undernutrition are rare^(^ ^17^ ^,^ ^31^ ^)^. The International Lipid-Based Nutrient Supplements Study Group (iLiNS) has previously reported that small-quantity LNS did not increase the physical activity of the children in the Prevention of Linear Growth Failure in Infants and Young Children With Lipid-based Nutrient Supplements (iLiNS-DOSE) trial^(^ ^31^ ^)^. However, the iLiNS-DOSE intervention was postnatal only, from 6 to 18 months of age, thus excluding the fetal period, which is crucial for brain growth and for later growth^(^ ^6^ ^)^ and development^(^ ^32^ ^)^ of children. The currently reported study (Supplementing Maternal and Infant Diet With High-energy, Micronutrient Fortified Lipid-based Nutrient Supplements; iLiNS-DYAD-M NCT01239693, https://clinicaltrials.gov/ct2/show/NCT01239693) was designed to assess the health impacts of small-quantity LNS given to mothers during pregnancy and 6 months postpartum and to their infants from 6 to 18 months of age^(^ ^33^ ^)^. Our hypothesis was that children in the intervention group would be more physically active at the age of 18 months than children in the control group.

## Methods

### Setting and participants

The study was conducted in a semi-urban and rural area of Mangochi District, Southern Malawi. This area has high prevalence of chronic infant undernutrition^(^ ^34^ ^)^. The activity sub-study was a part of a larger trial, iLiNS-DYAD-M, details of which have been published earlier^(^ ^35^ ^)^. In brief, iLiNS-DYAD-M was a randomised, single-blind, parallel-group controlled trial testing the health effects of supplementing maternal diet during pregnancy and lactation and infant diet from 6 to 18 months of age with LNS. This study was conducted according to the guidelines laid down in the Declaration of Helsinki, and all procedures involving human subjects were approved by the College of Medicine Research and Ethics Committee, University of Malawi, and by the Ethics Committee of Pirkanmaa Hospital District, Finland. We performed the trial according to Good Clinical Practice guidelines. Written or thumb-printed informed consent was obtained from all subjects.

The maternal enrolment took place through the antenatal clinics in Mangochi district hospital, Malindi hospital and Lungwena health centre. We included mothers whose ultrasound scan confirmed pregnancy of ≤20 completed gestation weeks. Detailed maternal inclusion and exclusion criteria were published previously^(^ ^35^ ^)^. For the physical activity sub-study, we recruited all participants who came to the last clinic visit of the main trial at the age of 18 months when they were still receiving the intervention. Those children who had moved out of the study area or whose guardian did not give consent for the sub-study were excluded. Data collection was conducted between January 2013 and March 2014.

### Randomisation and blinding

The details of the randomisation procedure can be found in an earlier publication^(^ ^35^ ^)^. In brief, a researcher not involved in the data collection created randomisation slips in blocks of nine and sealed the slips in opaque, numbered envelopes. Eligible pregnant women were requested to choose one of the top six envelopes in a stack. The contents of the envelope indicated her participant number and group allocation.

We used single-masked procedures for the LNS intervention; that is, field workers who delivered the supplements knew which mothers were receiving LNS. The research assistants measuring activity and anthropometric outcomes were kept blinded to the group allocation until the end of data collection. The researchers doing the analyses were blinded until the data were cleaned and the statistical analysis plan published online (www.ilins.org and online Supplementary Material S1).

### Interventions

The trial included three study groups^(^ ^35^ ^)^, which were collapsed into two groups for the purpose of this analysis. Children in the LNS group received 20 g of small-quantity LNS (20 g LNS) daily from 6 to 18 months of age. Their mothers had received 20 g of LNS during pregnancy and lactation (20 g LNS-P&L), daily during pregnancy and for 6 months thereafter. Both the 20 g milk containing LNS and 20 g LNS-P&L included twenty-two vitamins and minerals, 10 g fat (including linolenic acid and *α*-linolenic acid) and 2·6 g protein^(^ ^33^ ^,^ ^36^ ^)^. The main ingredients of the LNS were peanuts, vegetable oil, milk powder, and a vitamin and mineral mix. The 20 g daily ration of LNS would provide the RDA of most micronutrients for a healthy, breast-feeding infant^(^ ^36^ ^)^. LNS was produced and packed in individual 20 g foil sachets by Nutriset S.A.S.

Children in the control group did not receive supplementation, but their mothers had received either (a) one capsule daily of Fe-folic acid until delivery (60 mg Fe+400 µg folic acid) and one daily tablet of Ca (200 mg), akin to placebo, from delivery to 6 months postpartum (original Fe-folic acid (IFA) group) or (b) one tablet of multiple micronutrients (containing Fe-folic acid and sixteen additional micronutrients^(^ ^36^ ^)^) daily through pregnancy and 6 months postpartum (original micronutrients (MMN) group). Because of the absence of difference in child outcomes between the original IFA and MMN groups in our previous analyses^(^ ^33^ ^,^ ^35^ ^)^, and because children in these groups received no intervention from 6 to 18 months, we collapsed the IFA and MMN groups into a single control group.

### Follow up

Data collectors made weekly home visits to collect information on supplement use and fortnightly visits to deliver the supplements. Guardians were advised to divide the daily LNS ration into two equal proportions, mixed with porridge. As a result of a new quality assurance procedure in the supplement production, there was a brief interruption in LNS delivery during the trial implementation. Because of this episode, 121 children missed receiving LNS for a period that ranged from 1 to 41 d between 1 August and 11 September 2012. Further details on this episode were published earlier^(^ ^35^ ^)^. Apart from malaria, which was treated with lumefantrine/artemether at the study clinic, the participants’ medical conditions were treated in Malawi’s national health system, with the study team reimbursing the participants for all medical costs.

### Measurement of outcomes

Physical activity was measured over 1 week with the ActiGraph GT3X+ (ActiGraph LLC), a small accelerometer that records accelerations in three different axes: vertical, antero-posterior and medio-lateral^(^ ^26^ ^,^ ^37^ ^)^. While at the clinic, research assistants instructed the guardians to secure the accelerometer on the child’s right hip using an elastic belt and to allow the child to wear the device continuously throughout the day and night and to remove it only if the child showed signs of discomfort.

### Measurement of covariates

At the maternal enrolment visit, trained anthropometrists measured the mothers’ weight with digital scales (SECA 874 flat scale; Seca GmbH & Co.) and height with stadiometers (Harpenden stadiometer; Holtain Limited). Research assistants obtained maternal age and years of schooling by interviewing mothers at the enrolment visit. Household food insecurity was assessed with the Household Food Insecurity Access Scale (HFIAS)^(^ ^38^ ^)^. On the basis of the length of follow up and the number of supplement doses delivered home and returned unused, the mean adherence (proportion of days when the children consumed LNS supplements) was 77 %^(^ ^33^ ^)^.

Children’s date of birth was verified by the research assistants who visited the child soon after birth^(^ ^35^ ^)^. At the clinic visits at 6 and 18 months of age, research anthropometrists measured participants’ weight in triplicate to the nearest 20 g using an electronic infant-weighing scale (SECA 381 baby scale; Seca GmbH & Co) and length to the nearest 1 mm using a high-quality length board (Harpenden Infantometer; Holtain Limited). We calculated length-for-age *z*-score (LAZ) and weight-for-length *z*-score (WLZ) using the WHO 2006 Child Growth Standards^(^ ^39^ ^)^. Research assistants observed the children to assess their ability to walk at 18 months of age. Guardians were interviewed on how much the children were being carried by others, and children who were reportedly being carried 1 h or more every day or almost every day were classified as carried.

### Data processing and analyses

We analysed data using Stata/IC software, version 12.1 (StataCorp.). We set the level of statistical significance at 0·05 for all analyses. All the analyses were based on the principle of modified intention to treat; that is, we included all participants randomly assigned in the analyses, with the exception that two participants whose group allocations were incorrectly transcribed and assigned during enrolment were included in the group corresponding to the actual intervention they received throughout the trial. We considered physical activity data to be missing if the actual onset of measurement was over 30 d from the planned date. There were two reasons for this choice: children were no longer receiving intervention at this stage and improved motor skills at older age could result in accelerometer readings different from those of children at 18 months of age. The data reduction was done similarly to our earlier study^(^ ^31^ ^)^: we excluded the first and the last days of measurement as incomplete days, night time between 20.00 and 05.00 hours, and strings of ≥20 min of zeroes^(^ ^11^ ^)^. Participants with ≥4 d^(^ ^27^ ^)^ with ≥6 h^(^ ^40^ ^)^ of accelerometer data were included in the analyses. We set epoch length at 15 s^(^ ^11^ ^,^ ^41^ ^,^ ^42^ ^)^.

We used daily mean vector magnitude (VM) counts/15 s as the main outcome. The VM counts were calculated by taking the square root of the sum of squared activity counts of each of the three axes. Secondary outcomes included mean vertical axis counts/15 s, percentage of time in moderate-to-vigorous physical activity (MVPA), percentage of time being sedentary and percentage of active children. We calculated mean VM and vertical axis counts/15 s by averaging mean counts/15 s of each day over all valid days for each of the participants. The percentage of time spent in MVPA was defined using validated cutoff points of vertical axis activity counts ≥419 counts/15 s^(^ ^25^ ^)^ and percentage of time being sedentary as vertical axis activity counts ≤48 counts/15 s^(^ ^25^ ^)^. The proportion of active children was calculated according to the guidelines of the US National Association for Sports and Physical Education as those children whose mean time in MVPA over all valid days was ≥90 min/d^(^ ^43^ ^)^.

We used Fisher’s exact test to test for differences in the rate of loss to follow up between groups. We tested the hypothesis that physical activity of infants in the intervention group would be greater than that of infants in the control group for each activity outcome using Student’s *t* test, and the hypothesis that a greater proportion of children in the intervention group would reach 90 min of MVPA/d with a log-binomial regression model. We also drew kernel density plots for each of the outcomes. As a secondary analysis for assessing the mean VM counts in the intervention and control groups, we built a regression model adjusting for eight pre-specified variables (LAZ at 6 months, WLZ at 6 months, sex, season of activity measurement, birth order, maternal education, maternal age and HFIAS score) and for child carrying (carried *v*. not). We also performed a sensitivity analysis by testing the differences in mean VM counts between the three original groups, LNS, IFA and MMN, with ANOVA.

The sample size was originally calculated in accordance with the main objective of the trial: 288/group to detect an effect size of 0·3 of LNS on child length. The sample size of about 190 LNS and 380 control participants for this sub-study offered about 80 % power to detect an effect size of 0·25 sd in continuous outcomes at 5 % two-sided type I error rate.

## Results

**Fig. 1:**
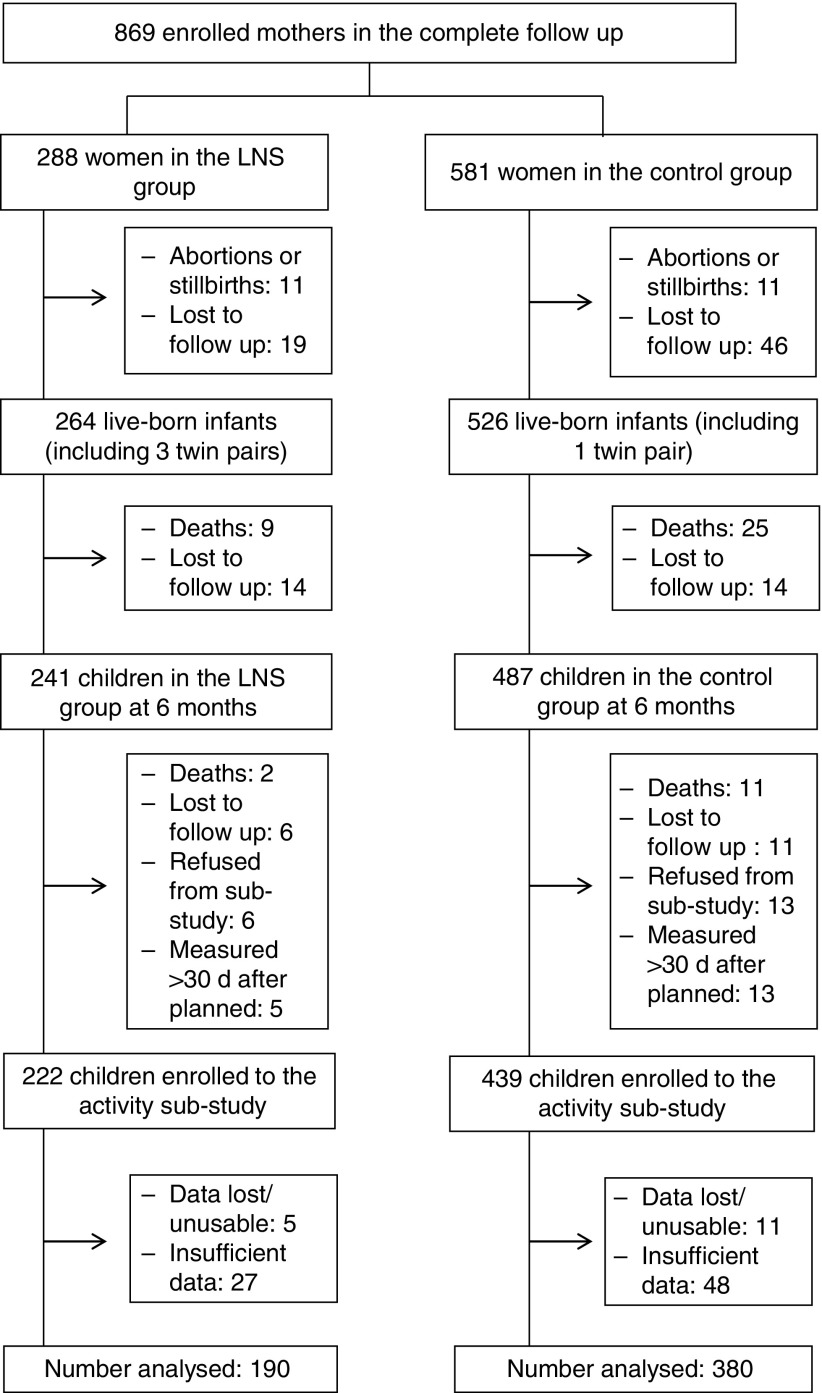
Participant flow. LNS, lipid-based nutrient supplements.

Fig. 1 presents the flow of the study participants and their mothers. Originally, 869 pregnant mothers were enrolled into this study. The 786 mothers who were still in the study at the time of delivery gave birth to 790 infants. Of the 661 children who were measured for activity at 18 months of age, 570 had sufficient data and were included in the activity analysis (78 % of the 728 children who were in follow up at the beginning of child intervention at 6 months of age).

Background characteristics of the children were similar in the intervention and control groups at 6 months of age (Table 1). Of the children who were in follow up at 6 months of age, 21 % in the LNS group and 22 % in the control group (*P*=0·85) were either lost to follow up or had insufficient accelerometer data. Children who were included in the analysis were generally similar in their mean background characteristics compared with the 158 children who were available at 6 months of age but were not included in the analysis, except that their mothers had fewer years of schooling. The included participants had a mean of 5·9 (sd 1·5, range 4–10) d of activity measurement with a median measurement time of 13·5 h/d (interquartile range 12·1–14·3 h).

**Table 1:** Background characteristics of participants and their mothers (Mean values and standard deviations; numbers and percentages)

	Control		LNS	
Variables	Mean	sd	Mean	sd
Situation at baseline, maternal enrolment				
Number of mothers	581		288	
Maternal age (years)	25	6	25	6
Maternal education, completed years of schooling	3·9	4	4·0	4
Maternal BMI (kg/m^2^)	22·0	2·6	22·2	3·1
Severely food insecure households				
*n*	208		104	
%	38		38	
Situation at 6 months, beginning of child intervention				
Number of children	487		241	
Males				
*n*	226		117	
%	46		49	
Length-for-age *z*-score	−1·25	1·2	−1·26	1·1
Weight-for-length *z*-score	0·39	1·2	0·36	1·1
Situation at 18 months, enrolment to physical activity measurement				
Number of children	439		222	
Age (months)	18·1	0·6	18·1	0·1
Season of activity measurement (%)	I: 28		I: 26	
	II: 20		II: 23	
	III: 28		III: 28	
	IV: 25		IV: 23	
Walking unassisted at 18 months (%)	95		97	
Children who were carried (%)*	17		17	

LNS, lipid-based nutrient supplements.

* Carried 1 h or more every day or almost every day by maternal report.

**Fig. 2:**
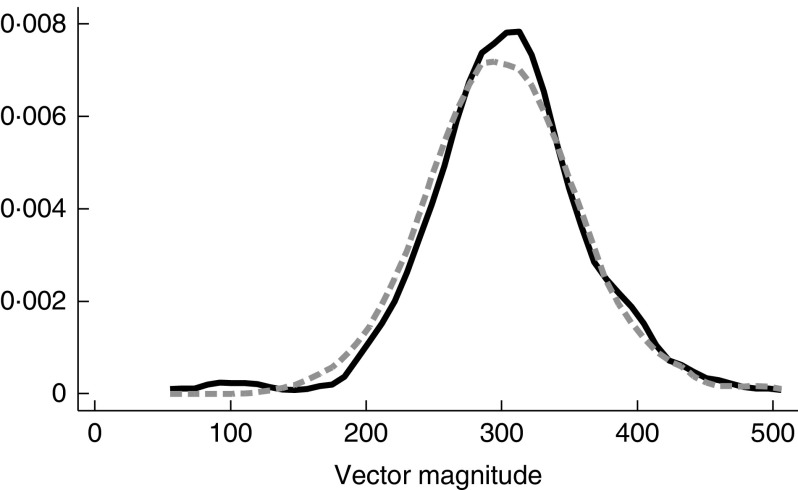
Kernel density plots of mean vector magnitude counts/15 s by groups. 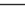, Lipid-based nutrient supplements; 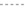, control.

The main outcome – mean VM accelerometer counts/15s – was 302 (SD 57) for all the included participants. The mean VM counts/15 s was 303 (sd 59) for children in the LNS group and 301 (sd 56) for children in the control group (*P*=0·65) (Fig. 2 and Table 2). There was no statistically significant difference in the secondary outcomes between the groups (Table 2 and online Supplementary Fig. S1). In the LNS group, 38·4 % of the children reached the recommendation of 90 min of MVPA/d, whereas the corresponding figure for the control group was 35·8 (risk ratio 0·93; 95 % CI 0·74, 1·17) %. The results from the regression analysis also showed no association between intervention and physical activity (*β*-coefficient 4·3; 95 % CI 15, 6; *P*=0·41). In the sensitivity analysis, comparing the three original groups, the mean VM counts/15 s was 303 (sd 59) in the LNS group, 306 (sd 62) in the MMN group and 297 (sd 49) in the IFA group (*P*=0·28).

**Table 2:** Physical activity at 18 months of age (Mean values and standard deviations; difference in mean values and 95 % confidence intervals)

	Trial group						
	Control (*n* 380)		LNS (*n* 190)		Comparison between the groups		
Variables	Mean	sd	Mean	sd	Mean	95 % CI	*P*
Vector magnitude (accelerometer counts/15 s)	301	56	303	59	2	−8, 12	0·65
Vertical axis (accelerometer counts/15 s)	140	32	144	36	3	−3, 9	0·27
Time in MVPA, by vertical axis (%)	11·1	3·5	11·4	4·0	0·3	−0·4, 0·9	0·38
Time sedentary, by vertical axis (%)	53·4	8·2	52·3	8·6	−1·1	−2·5, 0·3	0·13

LNS, lipid-based nutrient supplements; MVPA, moderate-to-vigorous physical activity.

## Discussion

The purpose of this sub-study of the randomised, controlled iLiNS-DYAD trial was to measure the effect of dietary supplementation with LNS, given to mothers during pregnancy and 6 months postpartum and to their infants from 6 to 18 months of age, on children’s physical activity in semi-rural Malawi. The mean accelerometer counts were slightly higher among the supplemented than among the non-supplemented participants in our sample, but the differences were small and statistically insignificant.

The results of this study are very similar to our previous findings where LNS did not increase the physical activity of Malawian children when given in 10–40 g quantities/d from 6 to 18 months of age^(^ ^31^ ^)^. In the current study, giving LNS to mothers during pregnancy and lactation did not seem to offer additional benefits in terms of physical activity of the children. We are not aware of any other studies measuring the effects of LNS supplementation on physical activity; however, there are previous studies examining physical activity after supplementation with multiple micronutrients^(^ ^12^ ^,^ ^44^ ^–^ ^46^ ^)^. These studies have demonstrated varying results. Several of them reported positive effects on physical activity from supplementation with either Zn^(^ ^47^ ^,^ ^48^ ^)^, multiple micronutrients^(^ ^46^ ^)^ or milk and multiple micronutrients in children with Fe-deficiency anaemia^(^ ^45^ ^)^. One of the studies found no effect^(^ ^44^ ^)^ and one reported mixed results^(^ ^12^ ^)^ of a milk-based intervention. All of the studies used observation as the method for measuring activity.

There are several theoretically plausible explanations for why the LNS intervention had no significant effect on children’s physical activity. One limiting factor could be relatively low (77 %) adherence to consuming LNS. The energy provided by LNS might replace rather than be additive to energy from breast milk or complementary foods. However, our previous studies suggest that provision of LNS does not reduce breast milk or complementary food intake at the age of 9–10 months^(^ ^49^ ^,^ ^50^ ^)^. According to the accelerometer measurement, children (even in the control group) were at least as active as has been reported for toddlers in high-income countries^(^ ^28^ ^,^ ^51^ ^)^, suggesting that they were already as active as might be expected at this age despite residing in a food-insecure area. There is considerable heterogeneity in the results of studies testing LNS for improving growth or treating moderate malnutrition^(^ ^52^ ^)^, implying that there could be underlying, context-specific mechanisms affecting children’s health and well-being and limiting response to nutrition interventions. Therefore, it is possible that nutrition interventions alone are not sufficient, but interventions that aim to improve children’s well-being also need to target other risk factors, such as infections^(^ ^7^ ^,^ ^53^ ^,^ ^54^ ^)^.

The main strengths of this study include the following: randomisation, which controls for confounders and decreases selection bias, objective measurement of physical activity, which eliminates reporting bias inherent to subjective physical activity measurement methods; and broad inclusion criteria, which increase the generalisability of results. The sample size was large enough to detect an effect size of 0·25 sd with about 80 % power.

The main weakness of the study was that the guardians could not be blinded to the treatment allocation of their children. However, because we used objective measurement of the outcome, this is unlikely to have affected the physical activity measurement. The weaknesses of accelerometers in measuring physical activity include the fact that they do not provide information on activity type or context, nor do they distinguish between the causes of movement, which may lead to overestimation of activity in infants who are frequently being carried by their caregivers^(^ ^11^ ^,^ ^18^ ^,^ ^55^ ^)^. However, overestimation of activity while the child is being carried appears to be more common among young infants who cannot walk^(^ ^18^ ^)^, whereas only 17 % of the 18-month-old children in both the control and intervention groups in our study were reportedly being carried ≥1 h daily or almost daily. Furthermore, we did not perform per-protocol analysis – that is, including only the most adherent children – because we did not have a placebo control and were thus unable to identify and exclude non-adherent children in the control group. Although loss to follow up was about 22 %, differences in background characteristics between participants who were enrolled or not enrolled into this sub-study were small, suggesting that attrition bias was not likely. Taking into account these shortcomings, we believe that the main results are valid and sufficiently representative of the target population, suggesting that LNS supplementation does not markedly increase the mean physical activity of young children in the defined target group.

In conclusion, our results do not support the hypothesis that LNS, given to mothers during pregnancy and 6 months postpartum and to their infants from 6 to 18 months of age, increases physical activity among 18-month-old children in semi-rural Malawi. Further research is needed to identify interventions that could improve the health and well-being of children who are living in food-insecure areas. Given the bidirectional relationship between nutrition and infections, whereby poor nutrition increases the risk for infections and infections can worsen nutritional status, these interventions might need to target both nutrition and infections. Furthermore, current recommendations for children’s physical activity are mainly based on parental-reported physical activity, a measure that is known to be biased. It would thus be important to establish recommendations for sufficient physical activity to support the health and development of children based on research using objective physical activity measures, such as accelerometers.

## Supplementary material

For supplementary material accompanying this paper visit http://dx.doi.org/10.1017/S0007114517000290. click here to view supplementary material

## References

[1] Adu-Afarwuah S, Lartey A, Brown KH, et al (2007) Randomized comparison of 3 types of micronutrient supplements for home fortification of complementary foods in Ghana: effects on growth and motor development. Am J Clin Nutr 86, 412–420. 17684213 10.1093/ajcn/86.2.412

[2] Adu-Afarwuah S, Lartey A, Okronipa H, et al (2016) Small-quantity lipid-based nutrient supplements provided to women during pregnancy and 6 mo postpartum, and to their infants from 6 mo of age, increases the mean attained length of 18-month-old children in semi-urban Ghana: a randomized controlled trial. Am J Clin Nutr 104, 797–808. 27534634 10.3945/ajcn.116.134692 PMC4997301

[3] Maleta KM, Phuka J, Alho L, et al (2015) Provision of 10-40 g/d lipid-based nutrient supplements from 6 to 18 months of age does not prevent linear growth faltering in Malawi. J Nutr 145, 1909–1915. 26063066 10.3945/jn.114.208181

[4] Mangani C, Maleta K, Phuka J, et al (2015) Effect of complementary feeding with lipid-based nutrient supplements and corn-soy blend on the incidence of stunting and linear growth among 6- to 18-month-old infants and children in rural Malawi. Matern Child Nutr 11, Suppl. 4, 132–143. 23795976 10.1111/mcn.12068 PMC6860208

[5] Iannotti LL, Dulience SJL, Green J, et al (2014) Linear growth increased in young children in an urban slum of Haiti: a randomized controlled trial of a lipid-based nutrient supplement. Am J Clin Nutr 99, 198–208. 24225356 10.3945/ajcn.113.063883 PMC3862455

[6] de Onis M, Dewey KG, Borghi E, et al (2013) The World Health Organization’s global target for reducing childhood stunting by 2025: rationale and proposed actions. Matern Child Nutr 9, Suppl. 2, 6–26. 10.1111/mcn.12075 PMC6860845 24074315

[7] Dewey KG & Mayers DR (2011) Early child growth: how do nutrition and infection interact? Matern Child Nutr 7, 129–142. 21929641 10.1111/j.1740-8709.2011.00357.x PMC6860756

[8] Hess SY, Abbeddou S, Jimenez EY, et al (2015) Small-quantity lipid-based nutrient supplements, regardless of their zinc content, increase growth and reduce the prevalence of stunting and wasting in young burkinabe children: a cluster-randomized trial. PLOS ONE 10, e0122242. 25816354 10.1371/journal.pone.0122242 PMC4376671

[9] Beckett C, Durnin JV, Aitchison TC, et al (2000) Effects of an energy and micronutrient supplement on anthropometry in undernourished children in Indonesia. Eur J Clin Nutr 54, S52–S59. 10.1038/sj.ejcn.1601005 10902987

[10] Manary MJ, Ndkeha MJ, Ashorn P, et al (2004) Home based therapy for severe malnutrition with ready-to-use food. Arch Dis Child 89, 557–561. 15155403 10.1136/adc.2003.034306 PMC1719944

[11] Cliff DP, Reilly JJ & Okely AD (2009) Methodological considerations in using accelerometers to assess habitual physical activity in children aged 0–5 years. J Sci Med Sport 12, 557–567. 19147404 10.1016/j.jsams.2008.10.008

[12] Jahari AB, Saco-Pollitt C, Husaini MA, et al (2000) Effects of an energy and micronutrient supplement on motor development and motor activity in undernourished children in Indonesia. Eur J Clin Nutr 54, 60–68. 10.1038/sj.ejcn.1601006 10902988

[13] Timmons BW, Naylor PJ & Pfeiffer KA (2007) Physical activity for preschool children – how much and how? Can J Public Health 98, Suppl. 2, 122–134. 18213943

[14] Timmons BW, Leblanc AG, Carson V, et al (2012) Systematic review of physical activity and health in the early years (aged 0–4 years). Appl Physiol Nutr Metab 37, 773–792. 22765840 10.1139/h2012-070

[15] Ekelund U, Luan J, Sherar L, et al (2012) Moderate to vigorous physical activity and sedentary time and cardiometabolic risk factors in children and adolescents. JAMA 307, 704–712. 22337681 10.1001/jama.2012.156 PMC3793121

[16] Grantham-McGregor S & Baker-Henningham H (2005) Review of the evidence linking protein and energy to mental development. Public Health Nutr 8, 1191–1201. 16277829 10.1079/phn2005805

[17] Faurholt-Jepsen D, Hansen KB, Van Hees VT, et al (2014) Children treated for severe acute malnutrition experience a rapid increase in physical activity a few days after admission. J Pediatr 164, 1421–1424. 24657125 10.1016/j.jpeds.2014.02.014

[18] Worobey J (2014) Physical activity in infancy: developmental aspects, measurement, and importance. Am J Clin Nutr 99, 729S–733S. 24477037 10.3945/ajcn.113.072397 PMC3927699

[19] Waterlow JC (1990) Energy-sparing mechanisms: reductions in body mass, BMR and activity: their relative importance and priority in undernourished infants and children In: Activity, Energy Expenditure and Energy. Requirements of Infants and Children, pp. 239–251 [B Schürch and NS Scrimshaw, editors]. Lausanne: International Dietary Energy Consultancy Group.

[20] Shetty PS (1999) Adaptation to low energy intakes: the responses and limits to low intakes in infants, children and adults. Eur J Clin Nutr 53, 14–33. 10.1038/sj.ejcn.1600741 10365978

[21] Aburto NJ, Ramirez-Zea M, Neufeld LM, et al (2009) Some indicators of nutritional status are associated with activity and exploration in infants at risk for vitamin and mineral deficiencies. J Nutr 139, 1751–1757. 19640971 10.3945/jn.108.100487

[22] Olney DK, Pollitt E, Kariger PK, et al (2007) Young Zanzibari children with iron deficiency, iron deficiency anemia, stunting, or malaria have lower motor activity scores and spend less time in locomotion. J Nutr 137, 2756–2762. 18029495 10.1093/jn/137.12.2756

[23] Lozoff B, Klein NK, Nelson EC, et al (1998) Behavior of infants with iron-deficiency anemia. Child Dev 69, 24–36. 9499554

[24] Yaméogo CW, Cichon B, Fabiansen C, et al (2017) Correlates of physical activity among young children with moderate acute malnutrition. J Pediatr 181, 235–241. 27866822 10.1016/j.jpeds.2016.10.073 PMC5282395

[25] Trost SG, Fees BS, Haar SJ, et al (2012) Identification and validity of accelerometer cut-points for toddlers. Obesity (Silver Spring) 20, 2317–2319. 22173573 10.1038/oby.2011.364

[26] Pulakka A, Cheung Y, Ashorn U, et al (2013) Feasibility and validity of the ActiGraph GT3X accelerometer in measuring physical activity of Malawian toddlers. Acta Paediatr 102, 1192–1198. 24102811 10.1111/apa.12412

[27] Hnatiuk J, Ridgers ND, Salmon J, et al (2012) Physical activity levels and patterns of 19-month-old children. Med Sci Sports Exerc 44, 1715–1720. 22543738 10.1249/MSS.0b013e31825825c4

[28] Wijtzes AI, Kooijman MN, Kiefte-de Jong JC, et al (2013) Correlates of physical activity in 2-year-old toddlers: the generation R study. J Pediatr 163, 791–799. 23523279 10.1016/j.jpeds.2013.02.029

[29] Johansson E, Hagstromer M, Svensson V, et al (2015) Objectively measured physical activity in two-year-old children – levels, patterns and correlates. Int J Behav Nutr Phys Act 12, 3. 25616495 10.1186/s12966-015-0161-0 PMC4312603

[30] Gardner JM & Grantham-McGregor SM (1994) Physical activity, undernutrition and child development. Proc Nutr Soc 53, 241–248. 8029231 10.1079/pns19940025

[31] Pulakka A, Ashorn U, Cheung YB, et al (2015) Effect of 12-month intervention with lipid-based nutrient supplements on physical activity of 18-month-old Malawian children: a randomised, controlled trial. Eur J Clin Nutr 69, 173–178. 25028082 10.1038/ejcn.2014.138

[32] Black MM, Walker SP, Fernald LCH, et al (2017) Early childhood development coming of age: science through the life course. Lancet 389, 77–90. 27717614 10.1016/S0140-6736(16)31389-7 PMC5884058

[33] Ashorn P, Alho L, Ashorn U, et al (2015) Supplementation of maternal diets during pregnancy and for 6 months postpartum and infant diets thereafter with small-quantity lipid-based nutrient supplements does not promote child growth by 18 months of age in rural Malawi: a randomized controlled trial. J Nutr 145, 1345–1353. 25926413 10.3945/jn.114.207225

[34] National Statistical Office Malawi and ICF Macro (2011) Malawi Demographic and Health Survey 2010. Zomba and Calverton, MD: National Statistical Office and ICF Macro.

[35] Ashorn P, Alho L, Ashorn U, et al (2015) The impact of lipid-based nutrient supplement provision to pregnant women on newborn size in rural Malawi: a randomized controlled trial. Am J Clin Nutr 101, 387–397. 25646337 10.3945/ajcn.114.088617

[36] Arimond M, Zeilani M, Jungjohann S, et al (2015) Considerations in developing lipid-based nutrient supplements for prevention of undernutrition: experience from the International Lipid-Based Nutrient Supplements (iLiNS) Project. Matern Child Nutr 11, 31–61. 10.1111/mcn.12049 PMC6860325 23647784

[37] John D & Freedson P (2012) ActiGraph and Actical physical activity monitors: a peek under the hood. Med Sci Sports Exerc 44, S86–S89. 22157779 10.1249/MSS.0b013e3182399f5e PMC3248573

[38] Coates J, Swindale A & Bilinsky P (2007) Household Food Insecurity Access Scale (HIFAS) for Measurement of Household Food Access: Indicator Guide (V.3). Washington, DC: Food and Nutrition Technical Assistance Project, FHI 360.

[39] World Health Organization (2006) WHO Child Growth Standards: Length/Height-for-Age, Weight-for-Age, Weight-for-Length, Weight-for-Height and Body Mass Index-for-Age: Methods and Development. Geneva: WHO.

[40] Trost SG, Pate RR, Freedson PS, et al (2000) Using objective physical activity measures with youth: how many days of monitoring are needed? Med Sci Sports Exerc 32, 426–431. 10694127 10.1097/00005768-200002000-00025

[41] Oliver M, Schofield GM & Kolt GS (2007) Physical activity in preschoolers: understanding prevalence and measurement issues. Sports Med 37, 1045–1070. 18027993 10.2165/00007256-200737120-00004

[42] Pate RR, O’Neill JR & Mitchell J (2010) Measurement of physical activity in preschool children. Med Sci Sports Exerc 42, 508–512. 20068498 10.1249/MSS.0b013e3181cea116

[43] National Association for Sport and Physical Education (2009) Active Start: A Statement of Physical Activity Guidelines for Children from Birth to Age 5, 2nd ed Sewickley, PA: American Alliance for Health, Physical Education, Recreation and Dance.

[44] Gardner JMM, Grantham-McGregor SM, Chang SM, et al (1995) Activity and behavioral development in stunted and nonstunted children and response to nutritional supplementation. Child Dev 66, 1785–1797. 8556899

[45] Harahap H, Jahari AB, Husaini MA, et al (2000) Effects of an energy and micronutrient supplement on iron deficiency anemia, physical activity and motor and mental development in undernourished children in Indonesia. Eur J Clin Nutr 54, 114–119. 10902994 10.1038/sj.ejcn.1601011

[46] Aburto NJ, Ramirez-Zea M, Neufeld LM, et al (2010) The effect of nutritional supplementation on physical activity and exploratory behavior of Mexican infants aged 8-12 months. Eur J Clin Nutr 64, 644–651. 20354559 10.1038/ejcn.2010.52

[47] Sazawal S, Bentley M, Black RE, et al (1996) Effect of zinc supplementation on observed activity in low socioeconomic Indian preschool children. Pediatrics 98, 1132–1137. 8951265

[48] Bentley ME, Caulfield LE, Ram M, et al (1997) Zinc supplementation affects the activity patterns of rural Guatemalan infants. J Nutr 127, 1333–1338. 9202087 10.1093/jn/127.7.1333

[49] Kumwenda C, Dewey KG, Hemsworth J, et al (2014) Lipid-based nutrient supplements do not decrease breast milk intake of Malawian infants. Am J Clin Nutr 99, 617–623. 24368436 10.3945/ajcn.113.076588

[50] Hemsworth J, Kumwenda C, Arimond M, et al (2016) Lipid-based nutrient supplements increase energy and macronutrient intakes from complementary food among Malawian infants. J Nutr 146, 326–334. 26740684 10.3945/jn.115.215327

[51] Van Cauwenberghe E, Gubbels J, De Bourdeaudhuij I, et al (2011) Feasibility and validity of accelerometer measurements to assess physical activity in toddlers. Int J Behav Nutr Phys Act 8, 67. 21703004 10.1186/1479-5868-8-67 PMC3132156

[52] Lenters L, Wazny K, Webb P, et al (2013) Treatment of severe and moderate acute malnutrition in low- and middle-income settings: a systematic review, meta-analysis and Delphi process. BMC Public Health 13, Suppl. 3, S23. 24564235 10.1186/1471-2458-13-S3-S23 PMC3847503

[53] Humphrey JH (2009) Child undernutrition, tropical enteropathy, toilets, and handwashing. Lancet 374, 1032–1035. 19766883 10.1016/S0140-6736(09)60950-8

[54] Gough EK, Moodie EE, Prendergast AJ, et al (2014) The impact of antibiotics on growth in children in low and middle income countries: systematic review and meta-analysis of randomised controlled trials. BMJ 348, g2267. 24735883 10.1136/bmj.g2267 PMC3988318

[55] Tsai SY, Burr RL & Thomas KA (2009) Effect of external motion on correspondence between infant actigraphy and maternal diary. Infant Behav Dev 32, 340–343. 19327842 10.1016/j.infbeh.2009.02.002 PMC2725759

