# Supplementary material for: Effect of 12-month intervention with lipid-based nutrient supplement on the physical activity of Malawian toddlers: a randomised, controlled trial
Source: Br J Nutr. 2017 Feb 28;117(4):511–8. doi: 10.1017/S0007114517000290 (PMC5426340; doi:10.1017/S0007114517000290)

Effect of 12-month intervention with lipid-based nutrient supplement on physical activity of Malawian toddlers: a randomised, controlled trial

A. Pulakka, Y. B. Cheung, K. Maleta, K. G. Dewey, C. Kumwenda, J. Bendabenda, U. Ashorn, P. Ashorn

Supplemental figure 1. Kernel density plots for the secondary outcomes: vertical axis counts/15 s, % time in moderate-to-vigorous physical activity and % time being sedentary.

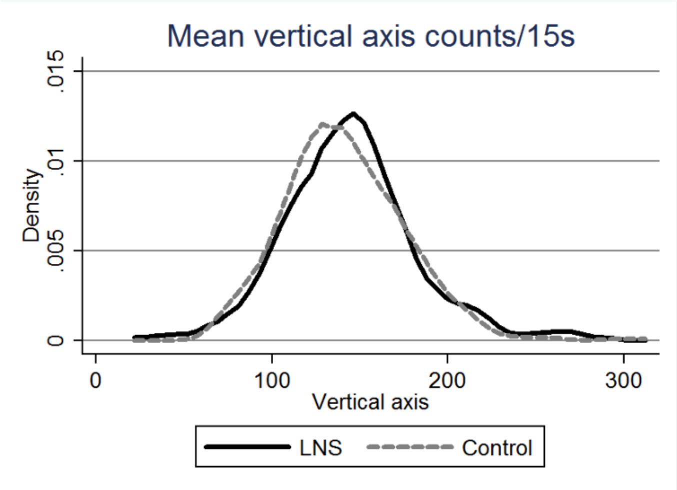

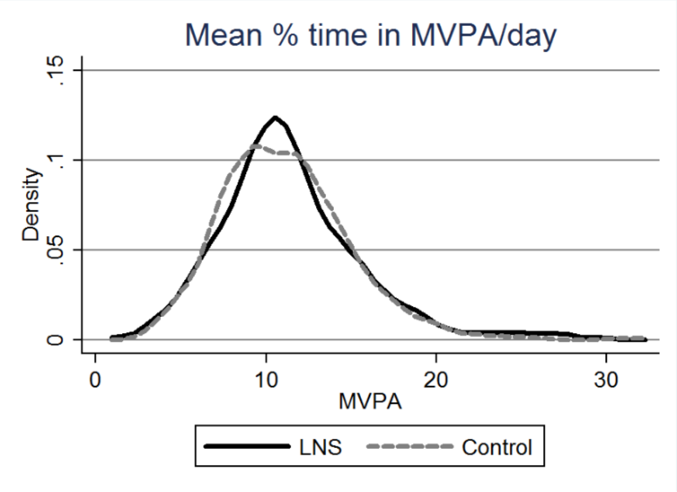

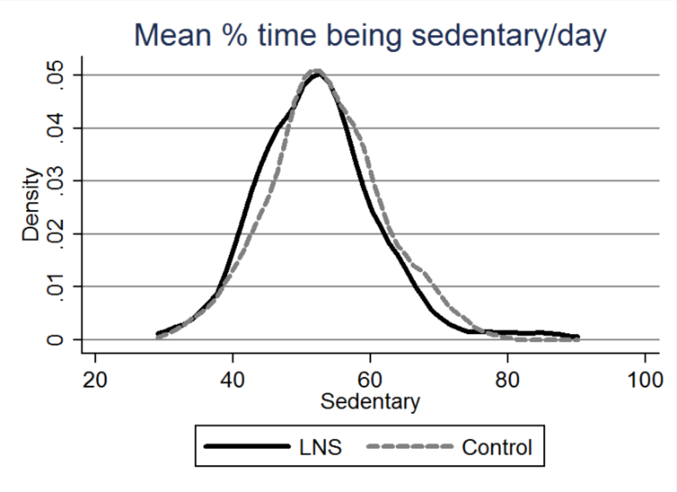

Supplement: Supplementary file 1 [file S0007114517000290sup001.zip › S0007114517000290sup001.pdf]
