# Supplementary material for: Effect of 12-month intervention with lipid-based nutrient supplement on the physical activity of Malawian toddlers: a randomised, controlled trial
Source: Br J Nutr. 2017 Feb 28;117(4):511–8. doi: 10.1017/S0007114517000290 (PMC5426340; doi:10.1017/S0007114517000290)
Supplement: Supplementary file 1 [file S0007114517000290sup001.zip › S0007114517000290sup002.pdf]

**Effect of 12-month intervention with lipid-based nutrient supplement on physical activity of Malawian toddlers: a randomised, controlled trial**

A. Pulakka, Y. B. Cheung, K. Maleta, K. G. Dewey, C. Kumwenda, J. Bendabenda, U. Ashorn, P. Ashorn

Supplemental File 1: Statistical Analysis Plan

**Prevention of Linear Growth Faltering in Infants and Young Children With Lipid-based Nutrient Supplements (iLiNS-DYAD)**

Statistical Analysis Plan

Appendix 25: The effect of LNS on physical activity (added on1.10.2015)

Table of Contents

1 Study objectives ........ 3
2 Hypotheses to be tested ........ 3
3 Definition of the physical activity outcomes ........ 3
4 Basis for the analysis: Intention to treat and per protocol ........ 4
5 Time points for the analyses ........ 4
6 Presentation of the study findings and hypothesis testing ........ 4
6.1 Success of enrolment, follow-up and physical activity measurement ........ 4
6.2 Baseline information ........ 4
6.3 Comparison of physical activity between the intervention and the control groups ........ 5
7 General notes on statistical methods ........ 5
7.1 Software ........ 5
7.2 Preparing physical activity data for analysis ........ 5
7.3 Preparing anthropometric data for analysis ........ 6
7.4 Multiple comparisons ........ 6
7.5 Confidence intervals ........ 6
7.6 Interaction and effect modification ........ 6
7.7 Covariate adjustment ........ 6
8 References ........ 7
9 Legends to the figures ........ 7
10 Figures ........ 8
Figure 1. Participant flow ........ 8
Figure 2. Kernel density plots of mean vertical axis counts/15 s by groups ........ 9
11 Tables ........ 10
Table 1 Background characteristics of participants and their mothers at baseline and at physical activity measurement ........ 10
Table 2 Physical activity at the trial groups ........ 11

# 1 Study objectives

The trial has three sets of objectives, defined at various phases of the trial.

The originally defined objective is to determine whether LNS consumed by the woman during pregnancy and the first 6 mo of lactation, and by the child from 6-18 mo, improves foetal and child growth, micronutrient status and neuro-behavioral development to a greater extent than consumption of iron and folic acid (IFA) during pregnancy only, or a multiple micronutrient (MMN) tablet during pregnancy and the first six months of lactation. Description of the other two objectives is presented in the main analysis plan.

The aim of the secondary analyses described in appendix 25 is to assess the impact of LNS supplementation to the mothers during pregnancy and first 6 months postpartum and to the offspring from 6 to 18 months of age on physical activity of children at the age of 18 months. This will be done by comparing physical activity of all measured children in the "complete follow-up" group who received one of the three interventions:

a) lipid-based nutrient supplements (LNS group)

b) multiple micronutrient supplementation (MMN group)

c) iron and folic acid supplementation (IFA group)

The two latter groups (IFA and MMN) will be collapsed into a single control group.

A secondary aim is to explore possible effect modifiers of the impact of LNS supplementation on physical activity.

# 2 Hypotheses to be tested

Infants' mean physical activity and % of active infants will be greater in the group provided with LNS from 6 to 18 months of age, and to their mothers during pregnancy and first 6 months postpartum, than in the control group (i.e. who received either iron-folic acid or multiple micronutrient supplementation).

# 3 Definition of the physical activity outcomes

<u>Primary outcome: mean accelerometer counts</u>

Physical activity counts used in the analysis are vector magnitude counts, calculated by taking the square root of the sum of squared activity counts of each three axis. The mean counts/15 s of each day will be averaged over all valid days (i.e. days with minimum of 6 hours of data, see section 7.2. for details) to produce mean of means for each participant.

Data for physical activity will be considered missing if the actual measurement date was over 30 days after the target date.

Secondary outcomes

Mean vertical axis counts: For mean vertical axis accelerometer counts/15 s, mean counts of each day are averaged over all valid days and the average value is used in the analyses.

% time in moderate-to-vigorous physical activity (MVPA): Percentage of time spend in MVPA is averaged over all valid days and the averaged value (per participant) is used in the analysis. MVPA is defined as vertical axis activity counts ≥ 419 counts/15 s (Trost et al. 2011). Trost cut point for vertical axis is used to allow comparison with previous studies using that cut point and older models of accelerometers with only vertical axis readings.

% time being sedentary: Percentage of time spent being sedentary is averaged over all valid days and the averaged value (per participant) is used in the analysis. Sedentary time is defined as vertical axis activity counts ≤48 counts/15 s (Trost et al. 2011).

% of active children: Children, whose mean time in MVPA over all valid days is ≥90 minutes are considered active. Ninety minutes is based on the guidelines of U.S. National Association for Sports and Physical Education (NASPE 2009).

## 4 Basis for the analysis: Intention to treat and per protocol

The primary analysis will be by intention-to-treat, i.e. analysis according to original group assignment regardless of protocol violations**.** For assessing the success of the enrolment, all available data from participants lost to follow-up will be included.

## 5 Time points for the analyses

All the above analyses will be done at the end of the intervention when the child is 18 months old.

## 6 Presentation of the study findings and hypothesis testing

### 6.1 Success of enrolment, follow-up and physical activity measurement

All children in the "complete follow-up" who were not dropped out before 18 months of age were invited to participate in this sub-study. All enrolled participants and the success of their follow-up, including physical activity measurement, will be described in a flow chart (Figure 1). For additional information, drop-out rate (including participants for whom enough accelerometer data was not available) between groups will be tested with Fisher's exact test and baseline characteristics of drop-outs compared to those who completed the study will be tested with t-test or Fisher's exact test. P-values for these tests will be shown in the text.

### 6.2 Baseline information

Participant characteristics at birth and at physical activity measurement (at 18 months) will be tabulated by treatment arms as indicated in Table 1. Baseline information will be tested for differences between groups to give additional information but p-values will not be presented

in Table 1 of the eventual manuscript. Methods used for hypothesis testing are indicated in Table 1.

### 6.3 Comparison of physical activity between the intervention and the control groups

Figure 2 will show the kernel density plots for the main outcome, mean vector magnitude accelerometer counts by groups. Supplemental figures will show the kernel density plots for the secondary outcomes. The group means and standard deviations for the main outcome, mean vector magnitude counts, and for the secondary outcomes: mean vertical axis counts, % of time spent in MVPA and % of time spent being sedentary as well as the number (%) of active children, will be presented as indicated in Table 2. The table will also tabulate the difference in activity outcomes and their 95% confidence intervals between the intervention groups.

The difference between the two groups will be tested with Student's t-test (model without covariates) and regression model (model with covariates) and null-hypothesis of no difference between groups will be rejected if $P<0.05$.

As a sensitivity analysis, we will compare the three original groups (LNS, IFA and MMN). The differences will be tested with ANOVA (model without covariates) and ANCOVA (model with covariates) and null-hypothesis of no difference between groups will be rejected if $P<0.05$.

## 7 General notes on statistical methods

### 7.1 Software

All analyses will be done in Stata/SE version 12. The WHO 2006 multi-centre growth standard will be used for age-and-sex standardization of weight, length (height), weight-for-height, MUAC and head circumference.

### 7.2 Preparing physical activity data for analysis

Data that was originally compiled by ActiLife software from ActiGraph GT3X+ devices, will be extracted and combined using the following procedure:

- .gt3x files will be converted to .agd files (with 3 axes and 15s epoch length) and exported into a .csv file consisting data from several participants in ActiLife software
- .csv files will be brought to Stata/SE software and transferred to .dat file.
- Strings of consecutive zeroes of 20 minutes or more as well as night time (between 8:00 p.m. and 5:00 a.m.) will be deleted.
- First and last day of measurement will also be deleted as incomplete days

The data is used for the analyses if the participant has minimum of 4 valid days of data, i.e. days with minimum of 6 hours of data after the above mentioned data reduction.

### 7.3 Preparing anthropometric data for analysis

The same as for the primary outcome analysis

### 7.4 Multiple comparisons

The same as for the primary outcome analysis

### 7.5 Confidence intervals

The same as for the primary outcome analysis

### 7.6 Interaction and effect modification

The following variables will be tested for interaction between the intervention group and the primary outcome (mean vector magnitude accelerometer counts). All tests will be done using the likelihood ratio test. The variables tested could logically modify the effect of the nutritional intervention on infancy and physical activity. Variables included (as continuous variables where possible) in this analysis include:

1. The participant's length-for-age (below / above sample median) at 6 months
2. The participant's weight-for-length (below/above sample median) at 6 months
3. The participant's sex
4. Season of activity measurement
5. Birth order (first-born or not)
6. Maternal education
7. Maternal age
8. Household food security (HFIAS)

If a statistically significant interaction ($p<0.1$) is found, the outcome analysis will be completed as stratified by the respective predictor variable.

### 7.7 Covariate adjustment

The main analysis, the results of which will be shown in tables and figures, will be completed without any covariate adjustments.

As a secondary analysis we will construct a regression model for physical activity, adjusting for the participant's sex, season of activity measurement, birth order, maternal education, maternal age, and household food insecurity.

As a sensitivity test for the latter analysis, we will use two alternative methods to build the regression model:

1. Stratifying the model by the effect modifiers.
2. Inclusion in the model of only those variables that are associated with physical activity (mean vector magnitude accelerometer counts) at $p<0.1$ level.

## 8 References

National Association for Sport and Physical Education (NASPE). Active start: A statement of physical activity guidelines for children from birth to age 5. 2nd Edition ed.
Sewickley, PA, USA: American Alliance for Health, Physical Education, Recreation and Dance; 2009.
Trost SG, Fees SF, Haar SJ, Murray AD, Crowe LK. Identification and Validity of Accelerometer Cut-Points for Toddlers. *Obesity* 2012; 20(11): 2317-2319

## 9 Legends to the figures

Figure 1. Participant flow

Figure 2. Box-Whisker plots of time in moderate-to-vigorous physical activity by groups

## 10 Figures

Figure 1. Participant flow

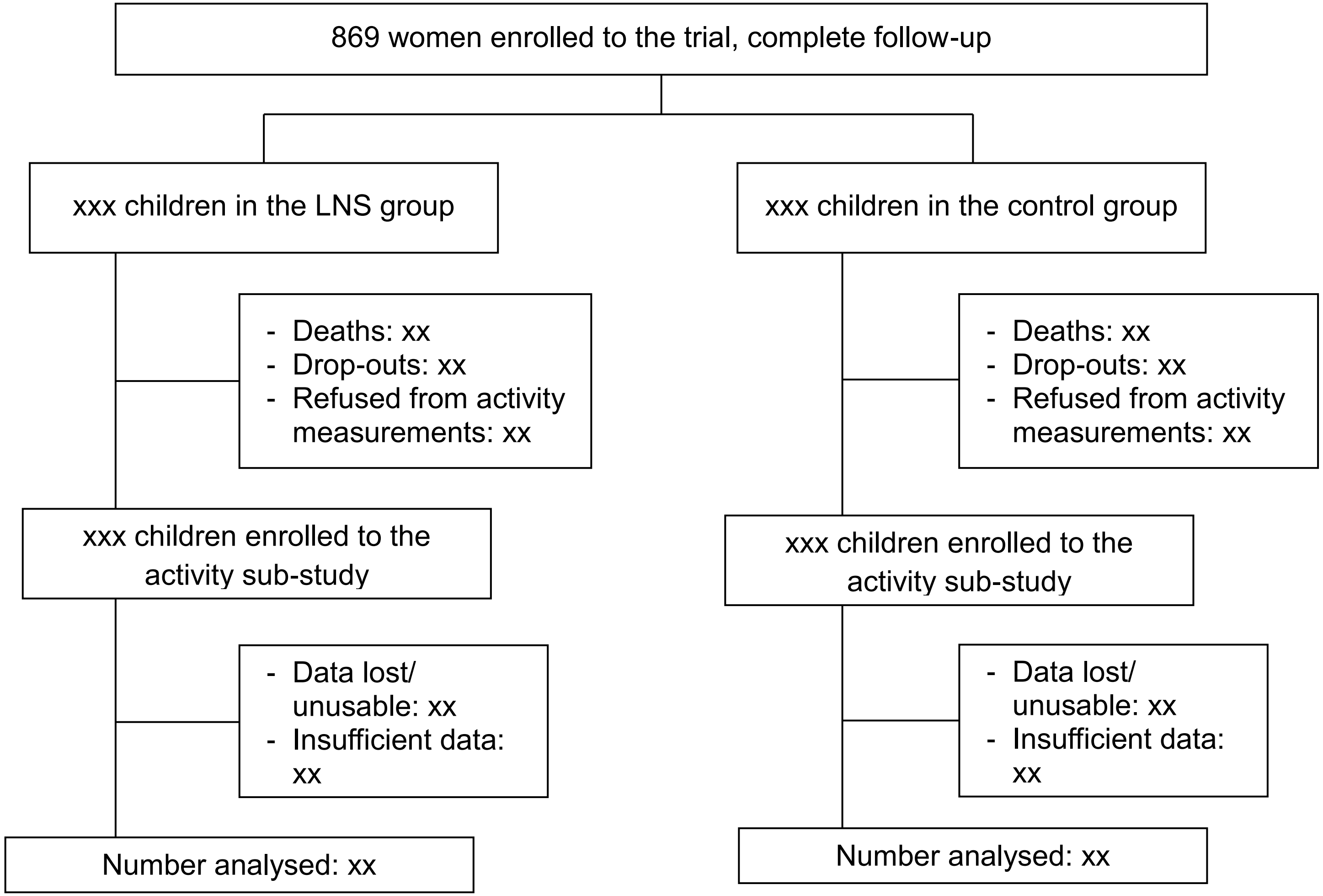

Figure 2. Kernel density plots of mean vertical axis counts/15 s by groups

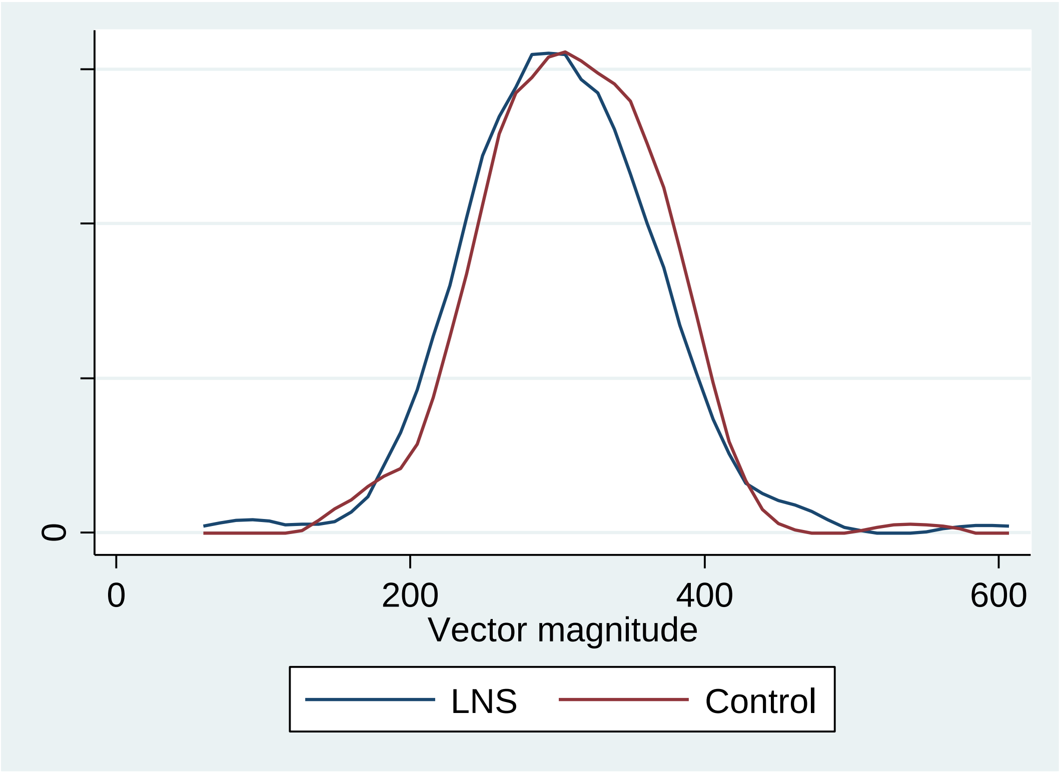

## 11 Tables

Table 1 Background characteristics of participants and their mothers at baseline and at physical activity measurement

| **Variable** | **LNS n=xxx** | **CONTROL n=xxx** | **Not enrolled n= xxx** | **Test** |
|---|---|---|---|---|
| **Situation at baseline (maternal enrolment)** | | | | |
| Mean (SD) maternal age, y | Xx (xx) | Xx (xx) | Xx (xx) | ANOVA |
| Mean (SD) maternal education, completed years of schooling | Xx (xx) | Xx (xx) | Xx (xx) | ANOVA |
| Mean (SD) maternal BMI ($kg/m^2$) | Xx (xx) | Xx (xx) | Xx (xx) | ANOVA |
| % of severely food insecure households | Xx | Xx | Xx | Fisher's exact test |
| **Situation at physical activity measurement** | | | | |
| Percentage of males | xx% | xx% | | Fisher's exact test |
| Mean (SD) age months | xx.x (xx.x) | xx.x (xx.x) | | Student's t-test |
| Season of activity measurement | I: xx.x%<br>II: xx.x%<br>III: xx.x%<br>IV: xx.x% | I: xx.x%<br>II: xx.x%<br>III: xx.x%<br>IV: xx.x% | | Fisher's exact test |
| Mean (SD) length-for-age z-score | xx.xx (xx.xx) | xx.xx (xx.xx) | | Student's t-test |
| Mean (SD) weight-for-length z-score | xx.xx (xx.xx) | xx.xx (xx.xx) | | Student's t-test |
| Walking unassisted | xx% | xx% | | Fisher's exact test |
| Mean (SD) minutes being carried/day | xx (xx) | xx (xx) | | Student's t-test |

Table 2 Physical activity at the trial groups

| | | | **Comparison between the groups** | |
|---|---|---|---|---|
| **Variable** | LNS | Control | Difference in means (95% CI) | P-value |
| Mean (SD) vector magnitude accelerometer counts/ 15 s | 303 (59) | 301 (56) | 3 (-8 to 12) | 0.65 |
| Mean (SD) vertical axis accelerometer counts/15 s | 144 (36) | 140 (32) | 3 (-3 to 9) | 0.27 |
| % of time in MVPA, by vertical axis | 11.4% | 11.1% | 0.3 (-0.4 to 0.9) | 0.38 |
| % of time sedentary, by vertical axis | 52.3% | 53.4% | -1.1 (-2.5 to 0.3) | 0.13 |
| % of children reaching recommendation of 90 min of MVPA/day | xx.x (x.x) | xx.x (x.x) | xx.x (x.x to x.x) | x.xxx |

LNS, lipid-based nutrient supplement; MVPA, moderate-to-vigorous physical activity; SD, standard deviation
